# Patient‐specific cutting guides increase accuracy of medial opening wedge high tibial osteotomy procedure: A retrospective case‐control study

**DOI:** 10.1002/jeo2.12013

**Published:** 2024-03-19

**Authors:** Jean‐Marie Fayard, Maxime Saad, Lucas Gomes, Sami Kacem, Hichem Abid, Thais D. Vieira, Pierre‐Jean Lambrey, Matthieu Ollivier, Mathieu Thaunat

**Affiliations:** ^1^ Ramsay Santé, Hôpital Privé Jean Mermoz—Centre Orthopedique Santy, FIFA Medical Center of Excellence Lyon France; ^2^ Department of Orthopedics and Traumatology, Aix Marseille University, APHM, CNRS, ISM, Sainte‐Marguerite Hospital Institute for Locomotion Marseille France; ^3^ Department of Orthopedics and Traumatology, St Marguerite Hospital Institute of Movement and Locomotion Marseille France

**Keywords:** 3D, medial open‐wedge high tibial osteotomy, patient‐specific cutting guide, tibial osteotomy accuracy

## Abstract

**Purpose:**

To compare the accuracy of patient‐specific guides (PSCG) to the standard technique in medial open‐wedge high tibial osteotomy (OWHTO). Secondary objectives were to evaluate factors that could influence accuracy and to compare the complication rate and operating time for both procedures.

**Methods:**

A retrospective analysis of prospective collected data was performed. Between March 2011 and May 2018, 49 patients with isolated medial knee osteoarthritis who were operated for OWHTO using PSCG and 38 patients using the standard technique were included. Preoperative and postoperative deformities were evaluated on long leg radiographs by measuring the mechanical medial proximal tibial angle, mechanical lateral distal femoral angle, hip knee ankle angle (HKA), and joint line convergence angle. Pre‐ and postoperative posterior tibial slope was also evaluated. Accuracy was evaluated by analysing the difference between the preoperative planned and the actual postoperative HKA. Operating time and complication rate were also recorded in both groups.

**Results:**

The mean preoperative HKA was 173.4° (±3.1°) in the PSCG group and 173.3° (±2.4°) in the standard group (*p* = 0.8416). Mean planned HKA were 182.8° (±1.1°) and 184.0° (±0°) respectively for the PSCG and the standard group. Mean postoperative HKA were 181.9° (±1.9°) and 182.6° (±3.1°) respectively for the PSCG and the standard group. An accuracy of ±2° in the HKA was achieved in 44 (90%) in the PSCG group and 24 (65%) in the standard group (*p* = 0.006). The probability of achieving a HKA accuracy was four times higher for patients in the PSCG group (odds ratio [OR] = 4.06, [1.1; 15.3], *p* = 0.038). Also, higher preoperative Ahlback grade was associated with precision, all other parameters being equal (OR = 4.2, [0.13; 0.97], *p* = 0.04).

**Conclusion:**

In this study, the PSCG technique was significantly more accurate for achieving the planned HKA in OWHTO. Complication rates and operating times were comparable between groups.

**Level of Evidence:**

Level IV, case‐control study.

**Abbreviations**
CAS: computer‐assisted surgery
HKA: hip knee ankle angle
HTO: high tibial osteotomy
ICC: intraclass correlation coefficient
JLCA: joint line convergence angle
mLDFA: mechanical lateral distal femoral angle
mMPTA: mechanical medial proximal tibial angle
OWHTO: open‐wedge high tibial osteotomy
PSCG: patient‐specific cutting guides

## INTRODUCTION

Valgus producing high tibial osteotomy (HTO) is a widely accepted procedure for treating medial osteoarthritis in varus knees [17, 21, 22, 26]. Medial open‐wedge high tibial osteotomy (OWHTO) is considered an excellent approach because it spares the fibular bone, has less risk of fibular nerve palsy, preserves bone stock, has less iatrogenic soft tissue damage, is technically less demanding and allows controlled correction in the sagittal and coronal planes [10, 13, 14, 23, 29].

Functional results are closely correlated with the correction of the lower limb's mechanical axis [1, 3, 6, 7, 9, 14]. Correction of this axis can be achieved by several methods, such as 3D patient‐specific cutting guides (PSCG), computer‐assisted surgery (CAS), and conventional radiography. The recent introduction of PSCG and navigation could result in better accuracy and therefore, better clinical outcomes [3, 26, 31].

The main objective of this study was to compare the accuracy of PSCG to the standard technique in medial OWHTO. The secondary objectives were to evaluate factors that could influence accuracy and to compare the complication rate and operating time for both procedures. The hypothesis was that 3D PSCG improves accuracy during OWHTO without additional complications.

## METHODS

A retrospective analysis was performed of prospectively collected data from our database between 2011 and 2018. A minimum of 2 years of follow‐up was required. The inclusion criteria were patients who had undergone OWHTO for treatment of symptomatic medial compartment knee osteoarthrosis (Ahlbäck ≤ 3) and had varus knee alignment (HKA < 180°), performed by the same single surgeon (Dr. Jean‐Marie Fayard), with complete radiological data. Exclusion criteria were combined femoral and tibial osteotomy and combined osteotomy and ACL reconstruction. Institutional review board approval (COS‐RGDS‐2022‐01‐003‐FAYARD‐JM) was granted for this study. Patient consent was collected, and no financial incentives were provided.

A consecutive series of patients who underwent OWHTO with 3D PSCG was compared to a series of patients who underwent OWHTO with the standard technique (i.e., Miniaci method) [18]. The two groups were comparable in terms of gender, age, weight, height, and preoperative deformity. A total of 87 patients were included in the study: 49 patients in the 3D PSCG group and 38 patients in the classic technique group.

**Figure 1:**
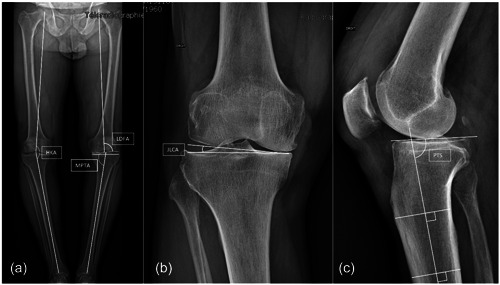
(a) Full‐length lower limb preoperative radiograph. Hip knee ankle angle was defined as the angle formed by the intersection of a line from the centre of the femoral head to the knee centre landmark and a second line from the centre of the ankle talus to the knee centre landmark. Lateral distal femoral angle was defined as the lateral angle between mechanical axis of the femur and the tangent line of the distal femoral condyles. Medial proximal tibial angle was defined as the medial angle between the mechanical axis of the tibia and the tangent line of the tibial plateaus. (b) Joint line convergence angle was defined as the lateral angle between the tangent line of the femoral condyles and the tangent line of the tibial plateaus. (c) Posterior tibial slope angle is formed between a line perpendicular to the longitudinal axis of the tibia and a line passing through the anterior and posterior high points of the tibial plateau.

Preoperative and postoperative radiological assessments were performed using weight‐bearing full‐length lower limb radiographs with AP and lateral views to determine the hip knee ankle angle (HKA), medial proximal tibial angle (MPTA), lateral distal femoral angle (mLDFA), joint line convergence angle (JLCA) and posterior tibial slope (PTS) (Figure 1). The measurements were performed by three independent surgeons to assess the interrater reliability.

### Preoperative planning for 3D PSCG technique

The planned correction was calculated by the surgeon using conventional radiographs. Subsequently, all patients underwent a CT scan. The surgeon filled out an order form for the engineer that specified the correction objectives in the frontal and sagittal planes through variations in the HKA angle. In the present study, no corrections were intended in the sagittal plane.

The CT scan protocol consisted of acquiring images centred on the femoral head, the knee (allowing 15 cm from the distal femur and the proximal tibia to be captured), and on the ankle. The slice thickness was 0.625 mm for the knee and 2 mm for the hip and ankle. Using the CT images, virtual 3D femur and tibia models (NewClip Technics®, Haute‐Goulaine, France) were created for both the deformed and contralateral leg. These models were processed to achieve the intended correction planned by the surgeon on the long‐leg standing X‐rays.

**Figure 2:**
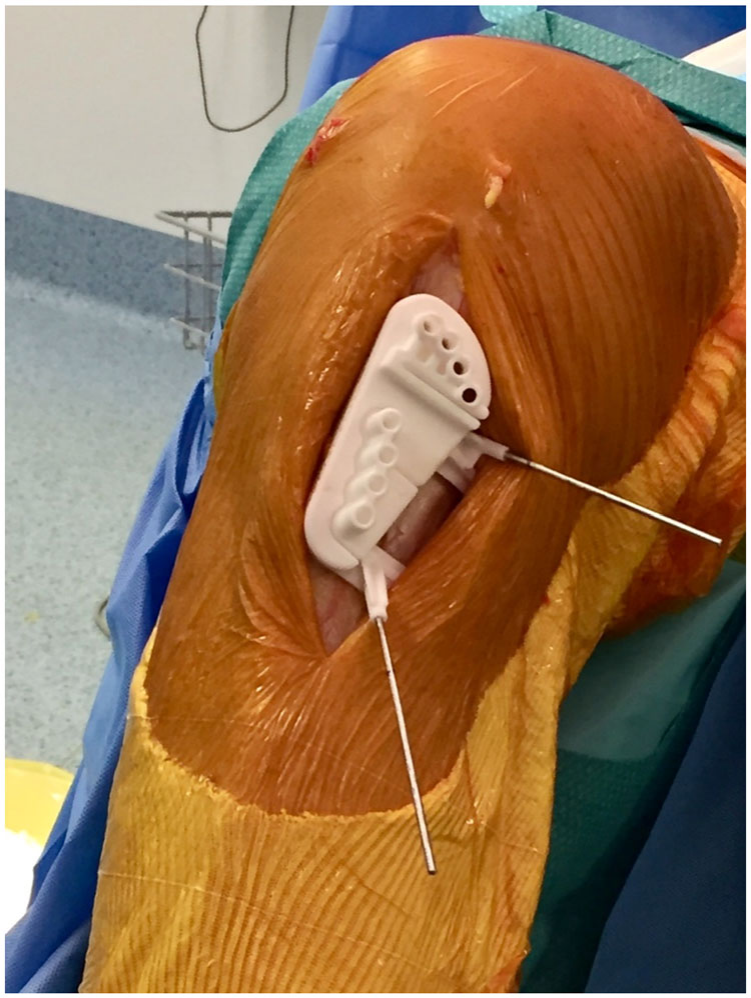
3D nylon® printed instrument.

Templates simulating the osteotomy and the PSCG were returned to the surgeon for final validation. Once approved, 3D nylon® printed instruments were made to achieve the planned correction (Figure 2).

#### Surgical technique

All patients had their procedure done by the same surgeon. They were placed in a supine position and had a tourniquet placed proximally on their thigh. In sequence, asepsis and antisepsis were made followed by sterile drape preparation.

A tibial anteromedial surgical approach was performed. The soft tissues were dissected to provide good exposure of the proximal tibia. The tourniquet was then deflated, and haemostasis was carried out.

The guide was positioned according to the template. The pegs in the nylon sterile cutting guide were placed in the posterior tibial cortex, according to plan. Then, two K‐wires (2.0 mm) were drilled into the landmarks on the template to secure the guide to the bone. Fluoroscopy was used to confirm the orientation of the osteotomy cut compared to the preoperative plan. An additional inferior 2.0‐mm K‐wire was drilled to protect the lateral cortex from hinge factures—the so‐called ‘golden pin’. The guide‐oriented holes needed for the plate were predrilled prior to performing the osteotomy with a 3.2‐mm drill bit.

The osteotomy was then performed through the PSCG in place. Once the cut was completed, only the golden pin was left in place. The osteotomy was then gradually distracted with a lamina spreader until the predrilled screw holes matched the plate holes.

Once again, fluoroscopy was used to compare the OWHTO performed with the template. Then, the locked plate was positioned according to the predrilled holes. The bone defect was filled with a femoral head wedge allograft.

### Preoperative planning for the standard technique

Preoperative planning was performed on the preoperative full‐length lower limb radiographs using the Miniaci technique [18] to evaluate the angle at the osteotomy site.

#### Surgical technique

A single 2.0‐mm K‐wire was positioned free‐hand from the medial tibial cortex towards the tip of fibular head to simulate the osteotomy site. Fluoroscopy was used to check the K‐wire's orientation. Osteotomy was then performed just below the K‐wire. Progressive opening was done with fluoroscopy guidance and the desired correction angle at the osteotomy site was checked. As soon as the opening was complete, a locking plate (TOMOFIX®) was secured to the tibia.

In both groups, wound closure was done with absorbable suture in subcutaneous tissue and intradermic absorbable suture in the skin. A drain was left in place during the first 24 h. Postoperative management included progressive partial to full weightbearing with crutches as tolerated at Day 1 if the lateral hinge remained intact. In case of lateral hinge fracture, patients were nonweightbearing for 45 days.

### Outcome measures

The main outcomes for this analysis were final HKA, final mPTA, final PTS, accuracy in intended correction, length of the surgical procedure and complications. Accuracy was defined as the difference between the correction obtained on postoperative radiographs and the desired HKA (defined preoperatively), with an accepted range of ±1°. For patients in PSCG group, accuracy was considered as the desired correction ±1°. For patients in standard group, accuracy was considered as 184° ± 1° [28].

### Demographic data

Overall, 87 patients made up the study population: 49 patients in the PSCG group and 38 in the standard group. The demographic characteristics are reported in Table 1.

**Table 1:** Demographics of the analysed population.

	Patient‐specific cutting guides group (*N* = 49)	Standard technique group (*N* = 38)	Analysed population (*N* = 87)	*p*‐Value
Age (in years), *N*	49	38	87	0.1896 (Student)
Mean (SD)	55.2 (8.5)	53.2 (4.7)	54.3 (7.2)	
Median (IQR)	57.0 (51.0–61.0)	54.4 (50.7–57.2)	56.0 (50.7–59.0)	
Range	(34–70)	(41–59)	(34–70)	
Missing	0	0	0	
Age bracket, *N* (%)	49	38	87	
<50 years	11 (22.4)	9 (23.7)	20 (23.0)	
(50–60) years	23 (46.9)	29 (76.3)	52 (59.8)	
≥60 years	15 (30.6)	0 (0.0)	15 (17.2)	
Missing	0	0	0	
Sex, *N* (%)	49	38	87	0.4631 (*χ* ^2^)
Male	37 (75)	26 (68)	63 (72.4)	
Female	12 (24.5)	12 (31.6)	24 (27.6)	
Missing	0	0	0	
Alback Grade, *N*	49	38	87	0.0012
Mean (SD)	2 (0.5)	1 (0.4)	2 (0.5)	
Range	(1–3)	(1–3)	(1–3)	

The preoperative radiographic characteristics of each group are reported in Table 2.

**Table 2:** Preoperative radiographic characteristics of the analysed population.

	Patient‐specific cutting guides group (*N* = 49)	Standard group (*N* = 38)	Analysed population (*N* = 87)	*p*‐Value
Preop hip knee ankle angle (in °), *N*	49	38	87	0.4877 (Student)
Mean (SD)	173 (3.1)	173 (2.4)	173 (2.8)	
Median (interquartile range [IQR])	174 (171–176)	174 (171–175)	174 (171–176)	
Range	(167–180)	(169–178)	(168–184)	
Missing	0	0	0	
Preop medial proximal tibial angle (in °), *N*	49	38	87	0.1293 (Student)
Mean (SD)	85.3 (2.5)	86.1 (2.5)	85.7 (2.5)	
Median (IQR)	86 (84–87)	86.5 (84–88)	86 (84–87)	
Range	(75–89)	(82–93)	(75–93)	
Missing	0	0	0	
Preop posterior tibial slope (in °), *N*	49	38	87	0.5905 (Student)
Mean (SD)	85.8 (2.1)	86 (1.5)	85.9 (1.9)	
Median (IQR)	86 (85–87)	86 (85–87)	86 (85–87)	
Range	(80–90)	(83–89)	(80–90)	
Missing	0	0	0	
Preop lateral distal femoral angle (in °), *N*	49	38	87	0.0016 (Student)
Mean (SD)	89.2 (1.2)	90.1 (1.4)	89.6 (1.4)	
Median (IQR)	89 (89–90)	90 (89–91)	90 (89–90)	
Range	(86–92)	(87–93)	(86–93)	
Missing	0	0	0	
Preop joint line convergence angle, *N* (%)	49	38	87	0.6614 (*χ* ^2^)
0–2	30 (61.2)	25 (65.8)	55 (63.2)	
>2	19 (38.8)	13 (34.2)	32 (36.8)	
Missing	0	0	0	

Thirty patients (61%) had a JLCA ≤ 2 in the PSCG group and 25 (66%) in the standard group.

### Statistical analysis

Descriptive data analysis was conducted depending on the nature of the considered criteria. For qualitative data this included the number of filled and missing data and, for each modality, the frequency and percentage (referring to filled data). Proportions was estimated with their exact 95% confidence intervals (CI) when appropriate. Data were compared using the *χ*² test or Fisher's exact test, according to the expected values under the assumption of independence. For quantitative data, this included number of filled and missing data, arithmetic mean, standard deviation, median, first and third quartiles, minimum and maximum values. Data were made compared Student's *t*‐test or the Mann–Whitney–Wilcoxon test (nonparametric test comparing ranks) depending on the distribution of the variable of interest. Comparisons of paired data were made using Student's *t*‐test (parametric test) or Wilcoxon test (nonparametric test) depending on the distribution of the variable of interest.

The correlation between two quantitative variables was determined using the Pearson correlation coefficient because the variables were distributed normally. Multivariate logistic regression was used to measure the probability of having a HKA accuracy of ±2° achieved or not, with potential factors. All comparisons were performed with the level of statistical significance set at *p* < 0.05. All calculations were made with SAS for Windows (v 9.4; SAS Institute Inc).

## RESULTS

### Postoperative measurements

#### HKA

The mean desired HKA in the PSCG group was 182.8° (±1.1°) and 184.0° (±0°) in the standard group. The mean postoperative HKA was 182.0° (±1.7°) in the PSG group and 182.8° (±2.2°) in the standard group. The mean in accuracy (HKA planned—HKA postoperative) was −0.8 ° (±1.6°) in the PSCG group and −1.4° (±3.1°) in the standard group. When considering an accuracy of ±2°, 90% of patients were within the target range in the PSCG group and 65% in the standard group (*p* = 0.006) (Table 3).

**Table 3:** Accuracy of hip knee ankle angle (HKA) results according to reference value.

	Patient‐specific cutting guides group (*N* = 49)	Standard technique group (*N* = 38)	*p*‐Value
Postop HKA—planned HKA (in °), *N*	49	38	0.3334 (Student)
Mean (SD)	−0.8 (1.6)	−1.4 (3.1)	
Median (interquartile range [IQR])	−1 (−2 to 0)	−1 (−3 to 1)	
Range	(−7 to 2)	(−9 to 7)	
Missing	0	0	
Accuracy of ±2°, *N* (%)	49	38	0.006 (*χ* ^2^)
No	5 (10)	14 (35)	
Yes	44 (90)	24 (65)	
Missing	0	0	

#### PTS

Postoperatively, the mean PTS was 85.9° (±2.4°) in the PSCG group and 86.0° (±2.5°) in the standard group (*p* = 0.8812). No statistical difference was observed in the PTS correction between groups (*p* = 0.7325).

#### MPTA

The mean postoperative MPTA was 91.6° (±2.4°) in the PSCG group and 93.8° (±3.7°) in the standard group (*p* = 0.0026). The mean MPTA correction was 6.0° (±2.5°) in the PSCG group and 7.6° (±4.0°) in standard group (*p* = 0.0315).

#### LDFA

Postoperatively, the mean LDFA was 89.1° (±5.2°) in the PSCG group and 89.4° (±2.6°) in the standard group (*p* = 0.7460).

#### JLCA

Postoperatively, no modification of the JLCA was noted.

### Factors associated with accuracy

The adjusted logistic regression showed that the probability of achieving a postoperative accuracy of ±2° is significatively associated with the preoperative Ahlback grade. A higher preoperative Ahlback grade was associated with precision, all other parameters being equal (OR = 4.2, [0.13–0.97], *p* = 0.04) (Table 4).

**Table 4:** Multivariate analysis of factors associated with a hip knee ankle angle (HKA) accuracy of ±2°.

Within the precision range (−2; +2)	Odd ratio	SE	*p*‐Value	(95% confidence interval)
PSI guide	**4.07**	2.75	**0.038**	1.08–15.28
Body mass index > 25	2.33	1.50	0.190	0.66–8.22
Preoperative ahlback	**4.19**	3.04	**0.048**	1.01–17.36
Preoperative joint line convergence angle	0.76	0.19	0.273	0.46–1.24

*Note*: Bold values are statistically significant.

### Procedure duration

The mean procedure duration was 62.8 min in the PSCG group and 66.0 min in the standard group (*p* = 0.1657).

### Complications

In the PSCG group, there was one reported infection (2%), one case of nonunion at the last follow‐up (2%) and one proximal tibia fracture (2%). In the standard group, there were two cases of lateral cortex fracture (5%).

### Interrater reliability

**Figure 3:**
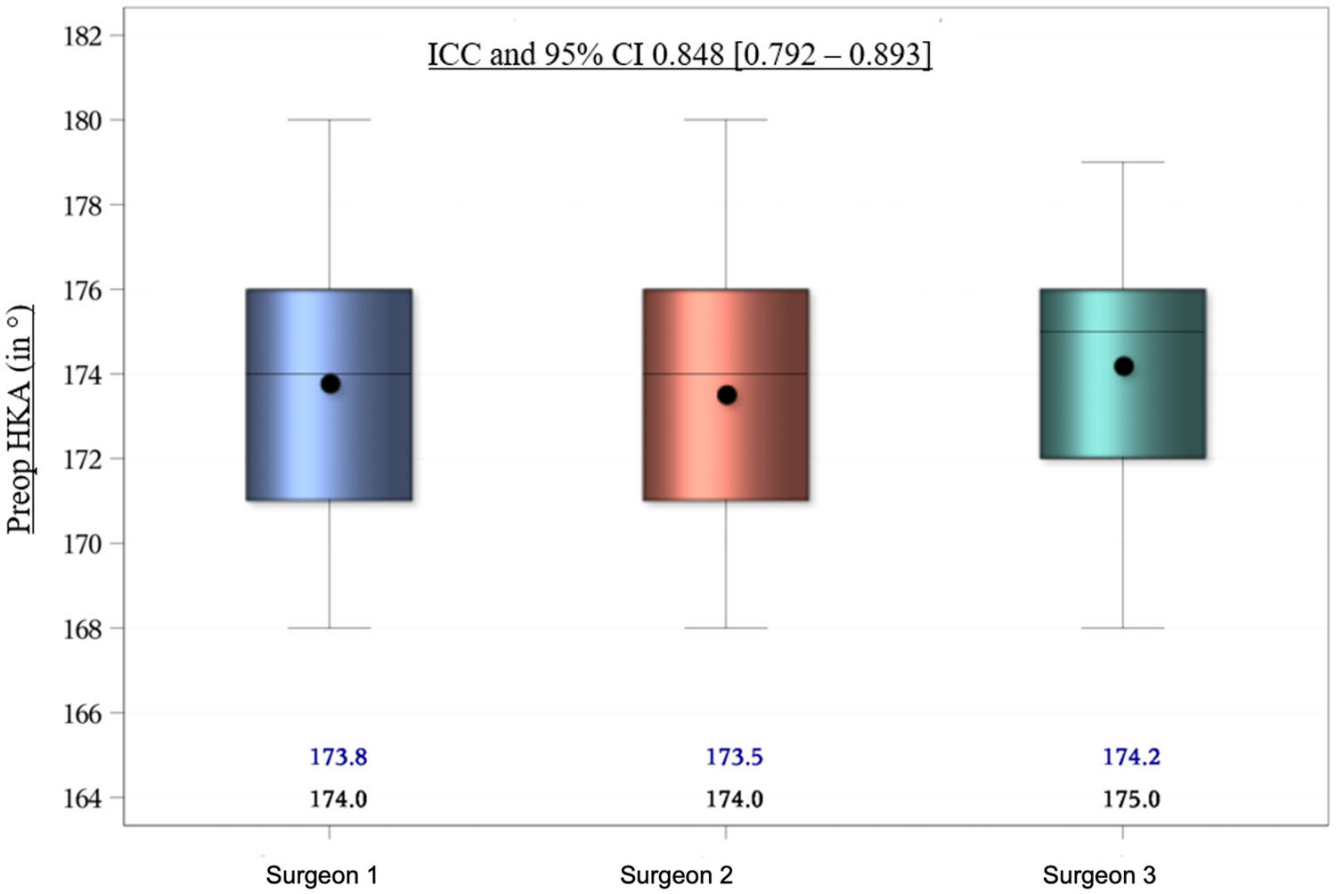
Inte‐rater intraclass correlation coefficient (ICC) for hip knee ankle angle (HKA) measured preoperatively. Cicchetti (1994) provides the following, often cited, guidelines for interpreting ICC measurements: Less than 0.40: poor/Between 0.40 and 0.59: fair/Between 0.60 and 0.74: good/between 0.75 and 1.00: excellent.

**Figure 4:**
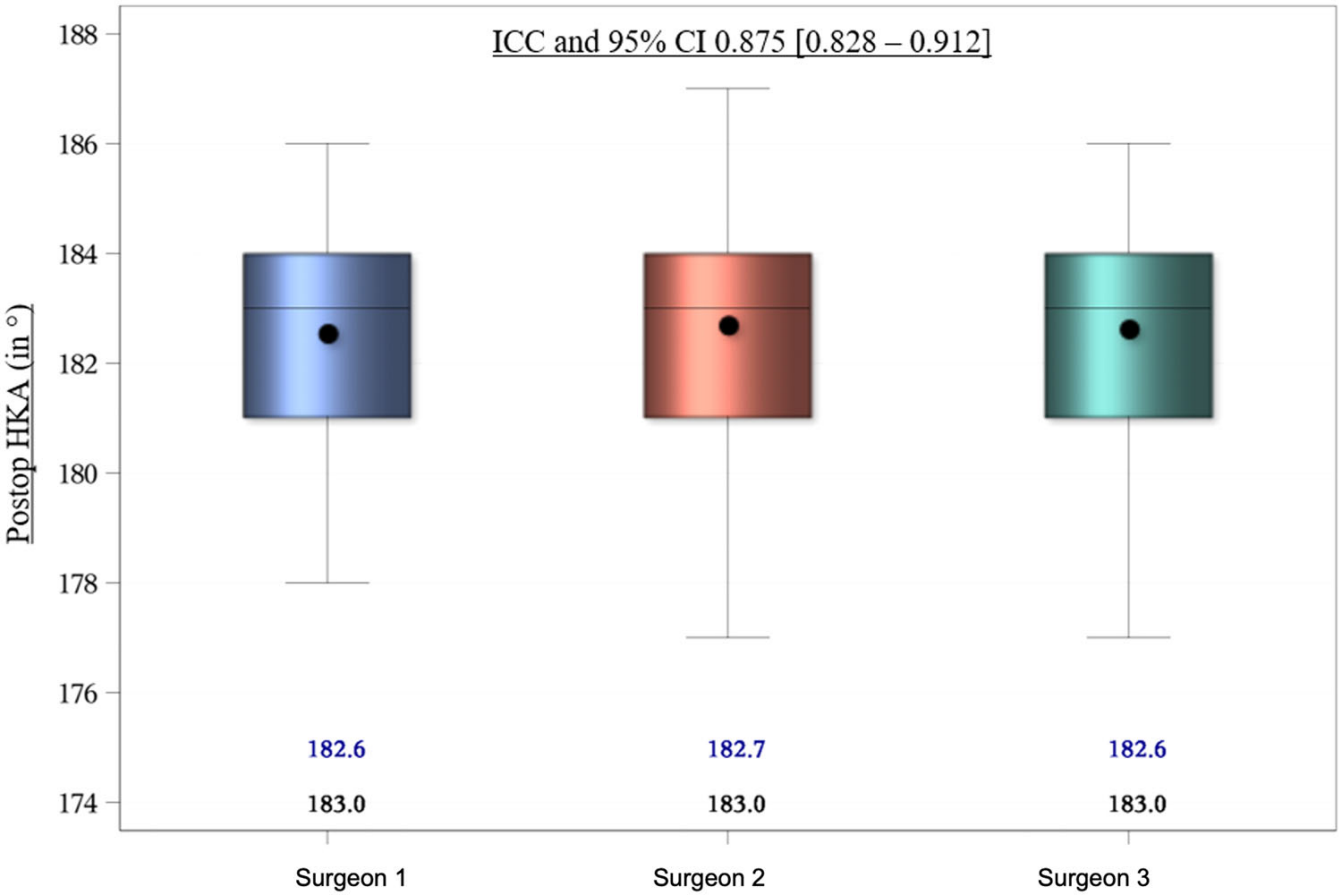
Interrater intraclass correlation coefficient (ICC) for hip knee ankle angle (HKA) measured postoperatively.

The intraclass correlation coefficient (ICC) and its 95% CI were calculated to measure the reliability of the different measurements between the three surgeon‐raters. ICC and 95% CI of the HKA preoperatively between the three raters was 0.848, which is excellent according to Cicchetti (Figure 3). ICC and 95% CI of the HKA postoperatively between the three raters was 0.875, which is excellent according to Cicchetti (Figure 4).

## DISCUSSION

The main finding of this study was that PSCG is more accurate than the standard method in OWHTO for achieving a desired HKA. Second, PSCG showed no additional complications and did not increase operating time compared to the standard method.

Recently, a literature review on accuracy of HTO reported that modern procedures seem to bear a surprisingly low accuracy with respect to the target angle [32]. There are few studies on PSCG accuracy [4, 8, 15, 19, 24, 33]. To our knowledge, this is the largest series comparing PSCG and standard technique accuracy; the 89.8% accuracy is comparable to the literature. Victor et al. [33] reported a mean deviation of 0.3° between the planned HKA and the postoperative HKA with PSCG, although their study had a small sample size (four OWHTO patients) and complex deformities within the inclusion criteria. Pérez‐Mañanes et al. [24] studied eight patients undergoing OWHTO with PSCG and found that the final axial correction achieved with PSCG was slightly better than the standard technique, although the difference between the two was not significant. The largest published PSCG study reported no significant differences between the desired correction and the correction obtained with a mean accuracy of 1 ± 0.9° for the HKA; however there was no comparison with a control group [4]. Saragaglia et al. [28] reported a 96% accuracy for a planned HKA angle of 184° ± 2° using navigation. PSCG results compare favourably with the accuracy achieved using CAS, without the high cost and the longer operating time associated with CAS [13].

Furthermore, in our study, there was a tendency to under‐correct the HKA: −0.8° (±1.6°) in the PSCG group and −1.4° (±3.1°) in the standard group. Previous authors have reported that under‐correction is common, and also, that it is more significant with opening‐wedge than closing‐wedge osteotomies [2, 20]. We believe this happens for two reasons: first, when using a 2D conventional radiographic technique, 3D surgical planning will likely over or underestimate a 2D‐measurement [27]; second, the bone deficit due to the saw blade thickness (~1.2 mm) might play a role [20]. Surgery duration is often discussed in modern OWHTO procedures. This study found a mean of 62 minutes for the PSCG group; there was no statistical difference between PSCG and standard techniques. Conversely, previous authors reported a 33% reduction in the operating time in the PSCG group compared to standard methods [24], along with a reduction in radiation exposure with PSCGs [34]. Surgery duration is mostly debated in navigated OWHTO; most authors suggest that the additional time required for navigation ranges from 10 to 30 min, potentially increasing the anaesthetic risk [25].

Complications related to PSCG OWHTO are similar those in the standard technique. Our study found an overall 5.7% complication rate and no difference between techniques. Lateral hinge fractures, infection and nonunion are the most frequent complications mentioned in the literature [11, 12]. A recent series of 209 patients reported an overall complication rate of 29.7% in OWHTO using a locking plate [12]. Undisplaced lateral hinge fracture was the most common adverse event (12%) [12]. Most of the series report no difference in the complication rate between PSCG and standard techniques [11, 19, 24, 33]. CAS techniques have complications related to trackers being fixed to the tibia and femur such as infection or fracture [16, 30]; one case of heterotopic ossification has also been described [5, 25].

The present study has several limitations, including a limited sample size and retrospective design with prospectively collected data. This was a historical study; thus, the preoperative plan was based only on the HKA correction. JLCA was not taken in account in either group during the planning process. Also, the postoperative measurement was done using a conventional 2D radiographic technique, which is known to be less precise than the preoperative 3D CT scan. The strengths of this study include the prospectively collected data and standardised diagnosis, treatment, and rehabilitation protocols.

## CONCLUSION

In this study, the PSCG technique was significantly more accurate than the standard method for achieving the planned HKA during OWHTO. Complication rates and operating times were comparable between groups. Our findings support PSCG techniques having fewer outliers than standard techniques and consequently, better accuracy. This is a determining factor for good results.

## AUTHOR CONTRIBUTIONS

*Substantial contributions to the conception or design of the work; or the acquisition, analysis, or interpretation of data for the work*: Jean‐Marie Fayard, Maxime Saad, Lucas Gomes, Sami Kacem, Hichem Abid, Thais D. Vieira, Pierre‐Jean Lambrey, Matthieu Ollivier and Mathieu Thaunat. *Drafting the work or revising it critically for important intellectual content*: Jean‐Marie Fayard, Maxime Saad, Lucas Gomes, Sami Kacem, Hichem Abid, Thais D. Vieira, Pierre‐Jean Lambrey, Matthieu Ollivier and Mathieu Thaunat. *Final approval of the version to be published*: Jean‐Marie Fayard, Maxime Saad, Lucas Gomes, Sami Kacem, Hichem Abid, Thais D. Vieira, Pierre‐Jean Lambrey, Matthieu Ollivier and Mathieu Thaunat. *Agreement to be accountable for all aspects of the work in ensuring that questions related to the accuracy or integrity of any part of the work are appropriately investigated and resolved*: Jean‐Marie Fayard, Maxime Saad, Lucas Gomes, Sami Kacem, Hichem Abid, Thais D. Vieira, Pierre‐Jean Lambrey, Matthieu Ollivier and Mathieu Thaunat.

## CONFLICT OF INTEREST STATEMENT

One or more of the authors has declared the following potential conflict of interest: Mathieu Thaunat is a paid consultant for, receives research support from, and has made presentations for Arthrex. Jean‐Marie Fayard is a paid consultant, receives research support and has made presentations for Arthrex, NewClip and Xnov. Matthieu Ollivier is a paid consultant, receives research support and has made presentations for Arthrex, NewClip and Stryker. The other authors declare no conflict of interest.

## ETHICS STATEMENT

Institutional review board approval (COS‐RGDS‐2022‐01‐003‐FAYARD‐JM) was granted for this study.

## Data Availability

Data available from Santy Orthopedic Center safeguarding.
